# Wearable sensors and machine learning fusion-based fall risk prediction in covert cerebral small vessel disease

**DOI:** 10.3389/fnins.2025.1493988

**Published:** 2025-02-19

**Authors:** Yuanyuan Zhou, Dingwen Zhang, Yingxiao Ji, Shuohan Bu, Xinzhu Hu, Congying Zhao, Zhou Lv, Litao Li

**Affiliations:** ^1^Department of Neurology, Hebei Medical University, Shijiazhuang, China; ^2^Department of Neurology, Baoding No.1 Central Hospital, Baoding, China; ^3^China National Clinical Research Center for Neurological Diseases, Beijing, China; ^4^Department of Neurology, Hebei General Hospital, Shijiazhuang, China; ^5^Department of Neurology, Hebei North University, Zhangjiakou, China; ^6^Department of Neurology, Handan Central Hospital, Handan, China; ^7^Hebei Provincial Key Laboratory of Cerebral Networks and Cognitive Disorders, Hebei General Hospital, Shijiazhuang, China

**Keywords:** wearable sensors, machine learning, cerebral small vessel disease, gait disturbances, fall risk

## Abstract

**Background:**

Fall risk prediction is crucial for preventing falls in patients with cerebral small vessel disease (CSVD), especially for those with gait disturbances. However, research in this area is limited, particularly in the early, asymptomatic phase. Wearable sensors offer an objective method for gait assessment. This study integrating wearable sensors and machine learning, aimed to predict fall risk in patients with covert CSVD.

**Methods:**

We employed soft robotic exoskeleton (SRE) to acquire gait characteristics and surface electromyography (sEMG) system to collect sEMG features, constructing three datasets: gait-only, sEMG-only, and their combination. Using Support Vector Machine (SVM), Random Forest (RF), Gradient Boosting Decision Tree (GBDT), and Neural Network (NN) algorithms, we developed twelve predictive models. Furthermore, we integrated the selected baseline data and imaging markers with the three original datasets to create three new integrated datasets, and constructed another twelve optimized predictive models using the same methods. A total of 117 participants were enrolled in the study.

**Results:**

Of the 28 features, ANOVA identified 10 significant indicators. The Gait & sEMG integration dataset, analyzed using the SVM algorithm, demonstrated superior performance compared to other models. This model exhibited an area under the curve (AUC) of 0.986, along with a sensitivity of 0.909 and a specificity of0.923, reflecting its robust discriminatory capability.

**Conclusion:**

This study highlights the essential role of gait characteristics, electromyographic features, baseline data, and imaging markers in predicting fall risk. It also successfully developed an SVM-based model integrating these features. This model offers a valuable tool for early detection of fall risk in CSVD patients, potentially enhancing clinical decision-making and prognosis.

## 1 Introduction

CSVD is a syndrome characterized by a series of pathological and imaging changes caused by various factors impacting the small arteries, veins, and capillaries within the brain (Mo et al., 2024; Pasi and Cordonnier, 2020; Ter Telgte and Duering, 2024). It constitutes 30% of strokes (Gorelick and Sorond, 2023; Kopczak et al., 2023) and can lead to dementia or mobility disorders (Duering et al., 2023; Dupré et al., 2024; Pasi and Cordonnier, 2020; Wardlaw et al., 2019; Zwanenburg and van Osch, 2017). Severe gait disorders can lead to falls (Sharma et al., 2023), increase the risk of hospitalization and disability, and shorten life expectancy (Mo et al., 2024; Mo et al., 2023).

Walking is a demanding activity that requires coordination of many different motor skills, cognitive abilities, as well as other elements. Numerous studies have confirmed the inextricable link between gait and cognition. Moreover, research has shown that dual-tasking leads to decreased walking speed and increased dual-task cost, and it provides a better prediction of dementia and a higher risk of falls compared to walking alone (Alzaid et al., 2022; Blumen et al., 2023; Ceïde et al., 2018; Rosso et al., 2019). In daily life, we often need to handle multiple tasks simultaneously, such as talking while walking or listening to music while running. Therefore, compared to walking alone, Dual-Task Walking (DTW) is regarded as a more accurate indicator of one’s ability to perform daily activities (McIsaac et al., 2018; Wajda et al., 2017).

Historically, owing to the constraints of evaluative methodologies, gait assessments have primarily focused on step speed (Markus and de Leeuw, 2023). However, advancements in wearable technology have led to the development of affordable and efficient sensors. These sensors, capable of capturing gait and sEMG data, enable the automatic and objective detection of gait impediments (Weizman et al., 2022). They are crucial for research in characterizing neurological gait patterns (Wuehr et al., 2020). SRE, a portable and lightweight mobility aid, can monitor gait parameters continuously during walking and aids in the recovery and endurance of impaired limbs (Awad et al., 2020; Bae et al., 2018; Slade et al., 2022; Xie et al., 2023). In addition, sEMG, a non-invasive and real-time monitoring technique, allows for the assessment of muscle activation, contraction, and fatigue during walking (Wu et al., 2024). This makes it a valuable tool in biomechanical studies. When integrated with machine learning (ML), sEMG becomes a promising method for the early detection and prediction of freezing of gait (FOG) in Parkinson’s disease patients (Elbatanouny et al., 2024; Yu et al., 2023).

CSVD gait disorders can lead to falls, and falls can increase the risk of hospitalization and mortality. In Europe, among the population aged 65 and above, there are 36,000 fatalities annually attributable to falls (Kancheva et al., 2024). The 2019 mortality data from the Chinese Center for Disease Control and Prevention indicate that falls are the second most common cause of death following traffic accidents, ranking 18th overall (Zhou et al., 2019). Moreover, approximately one-third of the residents aged 65 and older experience at least one fall per year, with some encountering multiple falls (Lewis et al., 2024). 30% of injuries that result from falls require medical attention, and 5% of falls result in fractures. Thus, accurately assessing gait function and promptly identifying high-risk CSVD patients prone to falls is imperative.

Currently, research on fall risk associated with CSVD is limited, mainly focusing on its correlation with imaging markers. Among these studies, a significant finding is the correlation between white matter hyperintensities (WMHs) and falls (Alzaid et al., 2022; Blumen et al., 2023; Crockett et al., 2021; Frisoni and van der Flier, 2023; Kancheva et al., 2024; Markus and de Leeuw, 2023; Mo et al., 2023; Sharma et al., 2023). A few studies have found that asymptomatic lacunar infarcts are associated with a higher risk of falls (Choi et al., 2012; Sharma et al., 2023). Several scholars have applied scales such as Berg Balance Scale and Short Physical Performance Battery (SPPB) to assess fall risk in older adults (Chan et al., 2024), but these methods have a certain degree of subjectivity. In recent years, with the advancement of digital technology, an increasing number of scholars have been using digital technologies like sensors and mobile applications to predict fall risk in the elderly (Koh et al., 2024). However, most of these studies capture data during single-task walking, which is not conducive to the early detection of the disease’s subtle symptoms.

Research has confirmed that covert CSVD without overt neurological manifestations is not silent (Mancuso et al., 2020; Wardlaw et al., 2021), and they often have mild neurological symptoms. To induce the hidden gait abnormalities of covert CSVD, this study continuously monitor spatial–temporal gait parameters and muscular electromyographic activities of covert patients during dual-task walking, employing sEMG and SRE. A fall risk prediction model for CSVD was constructed based on machine learning technology, with the aim of enabling early intervention, improved motor programs, and a decrease in fall risk.

## 2 Materials and methods

### 2.1 Participants

The data has been gathered at the Neurology Department of Hebei Provincial People’s Hospital since July 2023. For this study, the following inclusion and exclusion criteria were used to choose participants. Inclusion criteria: (1) Aged 50 years or above. (2) All participants underwent cranial MRI and satisfied the diagnostic standards of the Chinese Expert Consensus on the Diagnosis and Treatment of CSVD 2021 as well as the ESO Guideline on covert CSVD. (3) Be able to walk independently and can cooperate to complete gait assessments. Exclusion criteria: (1) Exist confirmed evidence of large artery atherosclerosis. (2) Residual motor deficits due to previous stroke. (3) Inability to walk because of unstable vital signs or comorbid serious illness. (4) Conditions such as articular deformity, lumbar spondylopathy, traumatic injuries and others that can affect gait or balance. (5) Cannot complete cognitive dual-task tests owing to severe visual and auditory impairments. (6) Taking medicine known to influence walking. All participants gave their informed consent to participate in the experiment, with ethical approval granted by the local authorities (NO.2023–420). The research was conducted in accordance with the standards of the Declaration of Helsinki.

### 2.2 Clinical baseline characteristics assessment

Comprehensive medical histories and blood biochemical data were collected from all research subjects, with the findings documented using a standardized evaluation protocol. Participants’ demographic profiles were compiled, encompassing details such as gender, age, height, body mass index (BMI), and educational duration. A thorough review of each individual’s medical history was conducted, focusing on conditions such as hypertension, diabetes, hyperlipidemia, coronary heart disease, and arrhythmias. Lifestyle factors, including smoking and alcohol consumption histories, were also recorded. The blood biochemical data were acquired, including levels of fasting blood glucose, glycated hemoglobin, OGTT2h, lipid profile, and homocysteine. During data collection, hypertension was defined as systolic blood pressure ≥ 140 mmHg and/or diastolic blood pressure ≥ 90 mmHg on three separate occasions without the use of antihypertensive medication, or as individuals with a previous diagnosis of hypertension who are on antihypertensive medication. Diabetes was defined as fasting blood glucose level ≥ 7.0 mmol/L, glycated hemoglobin≥6.5% or OGTT2h level ≥ 11.1 mmol/L, or as individuals with a previous diagnosis of diabetes who are using hypoglycemic medication. Hyperlipidemia was defined as having a previous diagnosis of hyperlipidemia and being on lipid-lowering medication, or meeting any of the following criteria: fasting lipid profile with triglyceride (TG) ≥1.7 mmol/L, total cholesterol (TC) ≥5.2 mmol/L, low density lipoprotein (LDL) ≥5.2 mmol/L, and low density lipoprotein (LDL) ≥5.2 mmol/L. lipoprotein (LDL) ≥3.4 mmol/L, and high-density lipoprotein (HDL) <1.0 mmol/L. Coronary heart disease was diagnosed in individuals with a previous diagnosis or those who met the diagnostic criteria outlined in the “Guidelines for the Diagnosis and Treatment of Stable Coronary Heart Disease 2018 Edition.” Arrhythmia was diagnosed in patients with a documented history of the condition or when an electrocardiogram performed during hospitalization indicated the presence of arrhythmia.

### 2.3 MRI collection

All participants in the study underwent 1.5 T MRI examinations (Signa HD, General Electric Company, USA).The imaging protocol encompassed a range of sequences, including T1-weighted imaging (T1WI), T2-weighted imaging (T2WI), fluid-attenuated inversion recovery (FLAIR), susceptibility-weighted imaging (SWI), diffusion-weighted imaging (DWI), and magnetic resonance angiography (MRA).The assessment of WMHs, cerebral microbleeds (CMBs), lacune, and enlarged perivascular spaces (EPVS) was conducted by two researchers who were unaware of the patients’ clinical information. In instances where there was a divergence of opinion, a third senior neurologist, who was also uninformed of the initial findings, provided an adjudicating assessment. The documentation of WMHs, lacune, CMBs, EPVS, and the total CSVD burden recorded according to the following criteria. WMHs refer to areas of abnormal signal within the white matter, which appear hyperintense on T2WI and FLAIR sequences. The Fazekas scale is utilized to evaluate periventricular WMHs (PWMHs) and deep WMHs (DWMHs) as follows. PWMHs: ① Grade 0: No abnormalities. ② Grade 1: Cap-like or pencil-thin lining lesions. ③ Grade 2: Smooth halo-like lesions. ④ Grade 3: Irregular lesions extending into deep white matter. DWMHs: ① Grade 0: No abnormalities. ② Grade 1: Punctate hyperintensities. ③ Grade 2: Beginning confluent hyperintensities. ④ Grade 3: Large confluent hyperintensities. Lacune manifest as round or oval fluid-filled cavities with signal intensities akin to cerebrospinal fluid, measuring 3 to 15 mm in diameter. Characteristically, these lesions demonstrate hypointensity on T1WI and hyperintensity on T2WI. Furthermore, on FLAIR sequences, lacunes are characterized by a central hypointense core encircled by a peripheral hyperintense rim. CMBs are characterized by punctate, round, or oval areas of signal void that possess well-defined margins when visualized on SWI (Duering et al., 2023). These lesions typically remain inconspicuous on FLAIR, T1WI, and T2WI sequences. According to the grading method by Lee S H and others, LI is graded based on quantity as follows: none, 0; mild, 1–3; moderate, 4–10; severe, >10. The dimensions of CMBs commonly range from 2 to 5 mm in diameter, with the largest not exceeding 10 mm. Based on the number of CMBs, they are classified into four grades: none, 0; mild, 1–5; moderate, 6–15; severe, >15. EPVS are characterized by fluid-filled intervals that envelop and parallel the trajectory of blood vessels, displaying signal characteristics analogous to those of cerebrospinal fluid, with a diameter typically measuring less than 3 mm. On MRI, EPVS demonstrate hypointensity on T1WI and FLAIR sequences, whereas hyperintensity is observed on T2-weighted imaging T2WI. We count the number of EPVS on one side of the brain with a higher prevalence and assess at least three sections. Based on the grading criteria established by the Edinburgh group, EPVS are classified into five levels according to their quantity: Level 0, no; Level 1, 1–10; Level 2, 11–20; Level 3, 21–40; Level 4, >40. The total CSVD burden is quantified by the aggregate score of four MRI markers. 1 point was assigned to each of the following markers: ① the presence of one or more lacunes; ② a Fazekas score of DWMHs ≥2 points and/or PWMHs ≥3 points; ③ the presence of one or more deep or infratentorial CMBs; ④ the presence of 11 or more basal ganglia EPVS. The total score ranges from 0 to 4 points. In this study, individuals with a total CSVD burden of 1 point or more are considered to have CSVD.

### 2.4 Test of Timed Up and Go (TUG)

The TUG test has gained widespread recognition as a valuable instrument for evaluating fall risk among the elderly (Williams and Nyman, 2021), demonstrates broad applicability across various clinical and research settings. Characterized by its ease of administration, simplicity, and robust reliability, the test effectively measures the ability to maintain balance and mobility during movement (Ortega-Bastidas et al., 2023). Endorsed by the American Geriatrics Society/British Geriatrics Society Clinical Practice Guidelines, the TUG has been recommended as an assessment to determine the fall risk in older individuals (Panel on Prevention of Falls in Older Persons, American Geriatrics Society and British Geriatrics Society, 2011). Furthermore, it has been proven that a cutoff time of 13.5 s for TUG test time has strong reliability, with a specificity of 100% and a sensitivity of 80% (Shumway-Cook et al., 2000).

**Figure 1:**
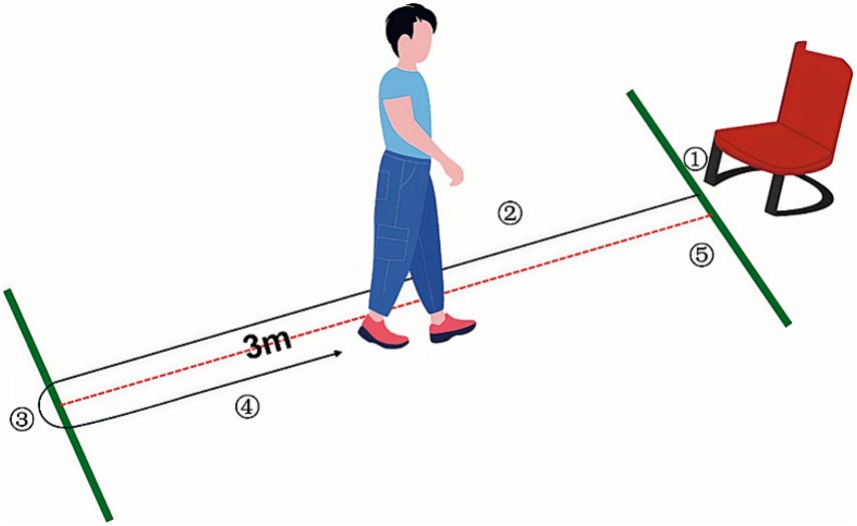
TUG test. ① Stand up. ② Walk forward 3 meters. ③ Turn back. ④ Return the walk. ⑤ Turn again and take a seat.

In this study, participants were instructed to sit on a chair with a seat back and wear their regular shoes, placing their feet on the marked tape positioned on the floor directly in front of the chair. Upon the evaluator’s command of ‘go,’ participants stood up and walked at their normal pace to the marked spot on the floor located 3 meters away from the chair. They then turned around, walked back to the chair, turned once again, and sat down, leaning against the backrest (Figure 1). No external physical help was permitted during the exam. The evaluator meticulously documented the time taken for participants to transition from standing away from the backrest to sitting down and leaning back against it. Before the formal test, participants were allowed to practice 1 to 2 times to ensure they understood the entire procedure. We considered TUG detection time ≤ 13.5 s as no fall risk and > 13.5 s as fall risk.

### 2.5 Data collection

#### 2.5.1 Materials

Gait assessment utilizes the Relink muscular exoskeleton (Yrobot Inc., Suzhou, China), a type of wearable SRE that can record and transmit gait information in real-time during walking, and then visualizes and analyzes the data multidimensionally through the Yrobot ANK software. In this experiment, gait data were collected by researchers from patients performing dual-task walking with the Relink muscular exoskeleton in its unpowered state. This approach facilitated the assessment of natural walking patterns, free from the influence of external support mechanisms typically found in powered exoskeletons.

**Figure 2:**
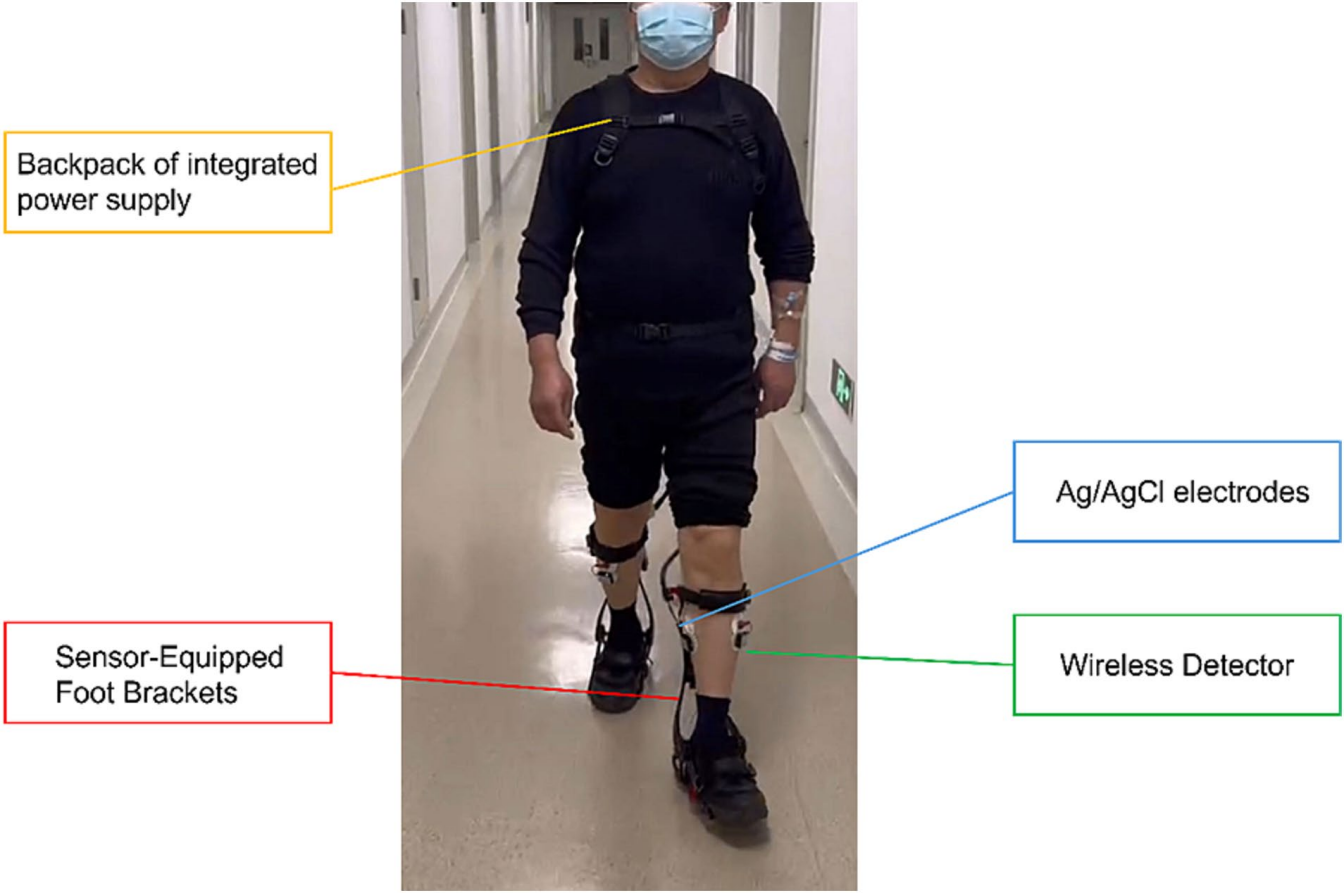
Configuration of the data acquisition platform. Gait characteristics during walking were acquired using the Relink muscle armor, and the signals were transmitted to the central processor on the backpack through sensors captured on the foot brackets. Subsequently, the data was analyzed and visualized using the Yrobot ANK software. sEMG characteristics were acquired using the 16-channel detector of the FREEEMG300 wireless sEMG system.

The FREEEMG300 Wireless Surface EMG System (BTS, Italy), operating at a sampling frequency of 1,000 Hz, comprises a laptop computer, a WIFI wireless receiver, an HP iPAQ hx4700 pocket PC, 16 Wireless EMG Probes, and several USB-PDA cables. It is capable of accurately collecting and analyzing the sEMG signals from the lower limbs during patient ambulation. Ag/AgCl electrodes (Xunda, Hangzhou, China) utilized in the test are disposable, with an adhesive area diameter of 52 mm and a conductive area diameter of 10 mm, ensuring optimal contact and signal transmission during the electromyographic data collection process. Figure 2 shows the Relink muscular exoskeleton, Ag/AgCl electrodes, and wireless detector used for data collection.

#### 2.5.2 Procedures

**Figure 3:**
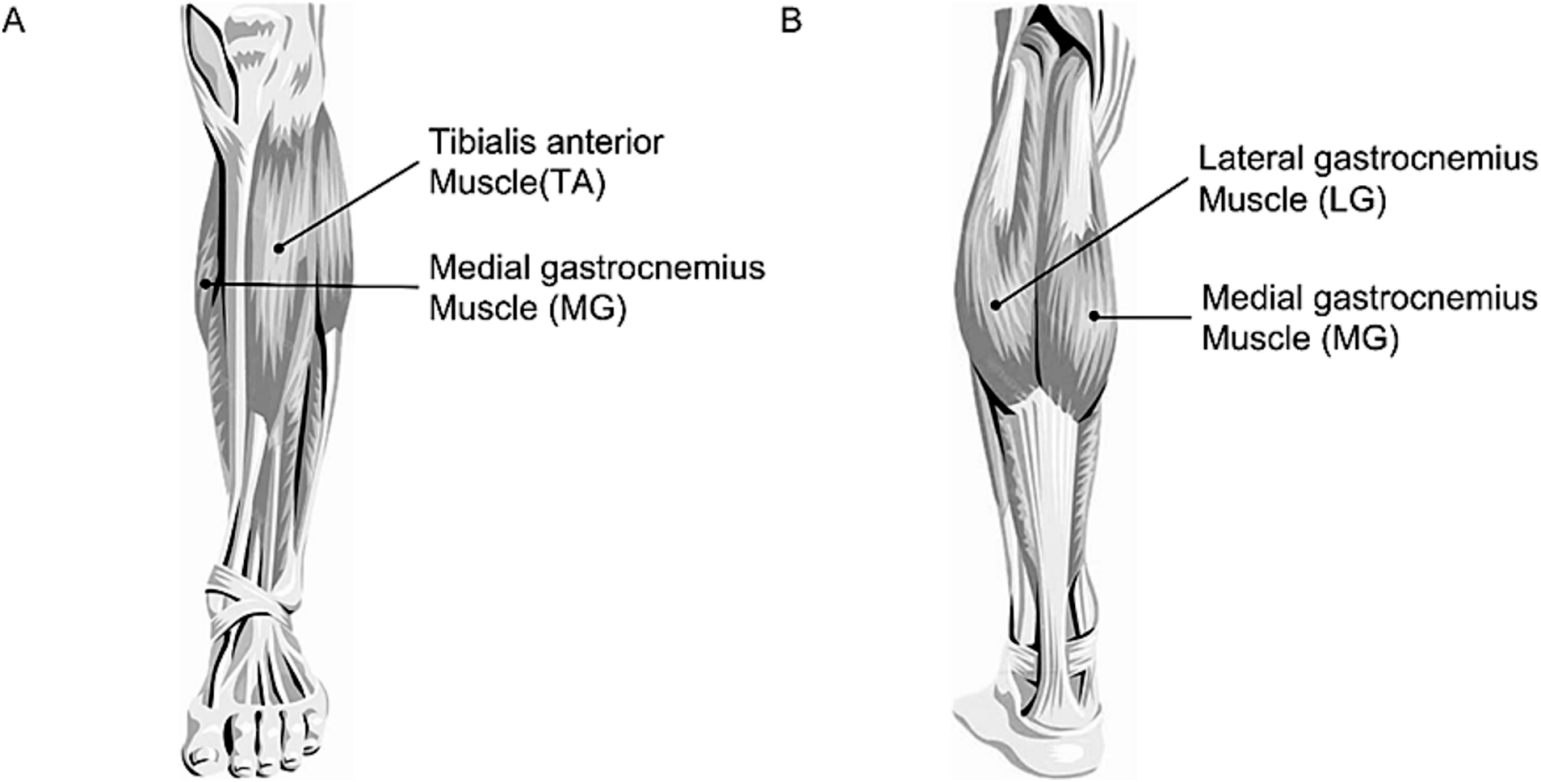
Location of sEMG detectors. sEMG signals were collected at the TA, MG, and LG muscles of both legs. **(A)** Lower limbs shown from the anterior. **(B)** Lower limbs shown from the posterior.

All participants were briefed on the experimental content and procedures, and preparatory activities were initiated once the patients had fully understood. The skin over the bilateral tibialis anterior (TA), medial gastrocnemius (MG), and lateral gastrocnemius (LG) was shaved, followed by abrasion and disinfection with 75% isopropyl alcohol to remove superficial dermal oils. Subsequently, the electrodes of the FREEEMG300 Wireless sEMG system were placed on the muscle bellies of TA, MG, and LG (Figure 3), then participants were assisted in donning the Relink Muscular Exoskeleton. The camera of the Wireless sEMG system was fixed at the starting point of the middle of the walking path to facilitate synchronized video recording, enabling identification of each gait cycle’s initiation and termination, and then the FREEEMG300 Wireless sEMG system and the Relink Muscular Exoskeleton were activated. Before the formal test, subjects were asked to walk within the designated area at their natural pace, to acclimatize them to the experimental procedure and to allow the evaluator to check that the Wireless sEMG system and Relink Muscular Exoskeleton were functioning properly. When all preparations were complete, formal data collection was initiated.

Participants were asked to walk at their usual pace along a corridor of about 25 meters in length, and to simultaneously complete consecutive subtractions of 7 from 500 without any pauses during the walking process. To avoid acceleration and deceleration effects, subjects were asked to take 3 steps before the test and another 3 steps after the test. To ensure the reliability of the collected data, at least 20 complete gait cycles within the effective camera range were captured, provided that the physical condition of the subjects allows. If subjects were physically unable to perform 20 gait cycles, they were guaranteed to complete at least 10 gait cycles; otherwise, their data were excluded.

#### 2.5.3 Data processing

Gait assessment involved the quantitative evaluation of critical kinematic parameters, including gait speed, cadence, stride length, and stance phase percentage. The Yrobot ANK software provided an advanced visualization tool, enabling the direct graphical representation of gait speed, cadence, and the stride lengths of both left and right limbs, as well as their respective stance phase percentages. The stride length and stance phase percentages were averaged for both limbs for statistical analysis. The sEMG signals were collected by the sEMG system during the walking process and were saved to a computer for further analysis. Stable gait cycles were selected from the walking process and the intensity of muscle contractions was assessed using time-domain metrics such as root-mean-square (RMS), integral electromyography (iEMG) and average electromyography (AEMG). To evaluate the degree of muscle fatigue, frequency-domain indicators such as Median Frequency (MF) and Mean Power Frequency (MPF) were employed. In this study, MATLAB R2023a was utilized to filter, rectify, and normalize the raw sEMG signals of the selected gait cycles, and to calculate the indicators for each gait cycle. Subsequently, the average value of each indicator was calculated for statistical analysis. The formulae for the indicators are as follows:

$$RMS=\sqrt{\frac{1}{T}\overset{T}{\underset{t=1}{\sum }}x^{2}\left(t\right)}$$

$\frac{1}{T}$ represents the reciprocal of the total time T.

$\overset{T}{\underset{t=1}{\sum }}$ indicates the summation from *t* = 1 to t = T.

$x^{2}\left(t\right)$ represents the squared value of the electromyography signal value at time point t.

$$\mathrm{iEMG}=\overset{N}{\underset{i=1}{\sum }}|x\left(i\right)|\Delta t$$

$|x\left(i\right)|:$ The absolute value of the electromyographic signal at the i-th sampling point.

Δt : The time interval between two adjacent sampling points.

$$\mathrm{AEMG}=\frac{1}{N}\overset{N}{\underset{i=1}{\sum }}x\left(i\right)$$

$\frac{1}{N}$ represents the calculation of the average value by dividing the total sum of the signal by the total number of sampling points N.

$\overset{N}{\underset{i=1}{\sum }}$ indicates the summation of all electromyographic signal values from the *𝑖* = 1 to *i* = N samples.

x(𝑖) represents the instantaneous value of the electromyographic signal at the 𝑖-th sampling point.

$$MF=\mathrm{frequency}\;\mathrm{at}\;\mathrm{which}\overset{MF}{\underset{0}{\int }}PSD\left(f\right)df=\frac{1}{2}\overset{\infty }{\underset{0}{\int }}PSD\left(f\right)df$$

PSD (𝑓): Power Spectral Density as a function of frequency f , delineating the distribution of signal power across the spectrum of frequencies.

df : Represents the differential of the integration variable f .

$$MPF=\frac{\int_{0}^{\infty }f\cdot PSD\left(f\right)df}{\int_{0}^{\infty }PSD\left(f\right)df}$$

### 2.6 Statistical analysis

We developed three distinct datasets leveraging the collected gait and sEMG characteristics: a dataset focused solely on gait, one on sEMG, and a comprehensive dataset integrating both. Variables with a *p*-value less than 0.05 were identified as significant predictors for fall-related analyses using the ANOVA chi-square test. Subsequently, SVM, RF, GBDT, and NN algorithms were applied to establish twelve predictive models based on the above three datasets. This study implemented a random division of the datasets into training and testing subsets with an 8:2 ratio, and evaluated the performance of the model using metrics such as AUC, sensitivity, specificity, accuracy, precision, recall and F1-score.Additionally, to further optimize the models, we integrated the selected baseline data and imaging markers with the three original datasets, creating three new integrated datasets. Subsequently, we constructed the models using the same methods. The modeling was performed with R software, version 4.4.0. The baseline characteristics of the participants were statistically analyzed using IBM SPSS Statistics for Windows, version 26.0 (IBM Corporation, Armonk, NY, United States).

## 3 Results

### 3.1 Baseline characteristics

**Figure 4:**
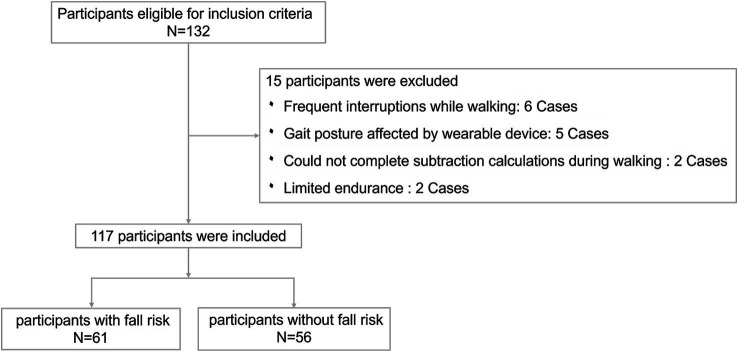
Flow diagram for selecting eligible patients.

The study enrolled 132 participants in total, depending on the inclusion and exclusion criteria. The data from 15 participants were excluded due to frequent interruptions during the walking process, gait posture affected by wearable devices, inability to cooperate in completing subtraction calculations, and limited endurance. The data of 117 participants were valid, consisting of 61 with fall risk and 56 without fall risk (Figure 4). Table 1 displays the baseline characteristics of these 117 participants. In our study, we compared two groups: those with fall risk and those without fall risk. Among the 14 baseline characteristics analyzed, we found significant differences (*p* < 0.05) in 3—age, height, and education duration—while the remaining 11 characteristics did not show significant differences.

**Table 1:** Baseline characteristics: comparison between patients with fall risk and those without fall risk.

Variables	No-fall risk	Fall risk	*χ2/F/Z*	*P*
Age(years)	61.00(13.00)	68.00(14.00)	−3.596	<0.001^***^
Height(cm)	168.13 ± 6.95	163.90 ± 7.15	0.284	0.002**
Weight(kg)	71.00(16.85)	69.00(15.00)	−2.456	0.014*
BMI(kg/m2)	25.95 ± 2.88	25.29 ± 3.05	0.209	0.233
Education duration(years)	9.00(4.00)	9.00(6.00)	−2.071	0.038*
Uric acid(μmol/L)	350.06 ± 72.46	298.47 ± 82.18	1.841	<0.001^***^
Homocysteine(μmol/L)	14.05(6.97)	14.30(11.15)	−0.420	0.674
Gender, n(%)			2.236	0.135
Male	37(66.1)	32(52.5)		
Female	19(33.9)	29(47.5)		
Smoking, n(%)			0.524	0.469
No	35(62.5)	42(68.9)		
Yes	21(37.5)	19(31.1)		
Drinking, n(%)			1.253	0.263
No	39(69.6)	48(78.7)		
Yes	17(30.4)	13(21.3)		
Hypertension, n(%)			2.991	0.084
No	20(35.7)	13(21.3)		
Yes	36(64.3)	48(78.7)		
Diabetes, n(%)			0.003	0.955
No	37(66.1)	40(65.6)		
Yes	19(33.9)	21(34.4)		
Hyperlipidemia, n(%)			0.743	0.389
No	24(42.9)	31(50.8)		
Yes	32(57.1)	30(49.2)		
Coronary Heart Disease, n(%)			0.301	0.583
No	48(85.7)	50(82.0)		
Yes	8(14.3)	11(18.0)		
Arrhythmia, n(%)			1.235	0.266
No	37(66.1)	46(75.4)		
Yes	19(33.9)	15(24.6)		

### 3.2 Model construction

#### 3.2.1 Variable selection

**Figure 5:**
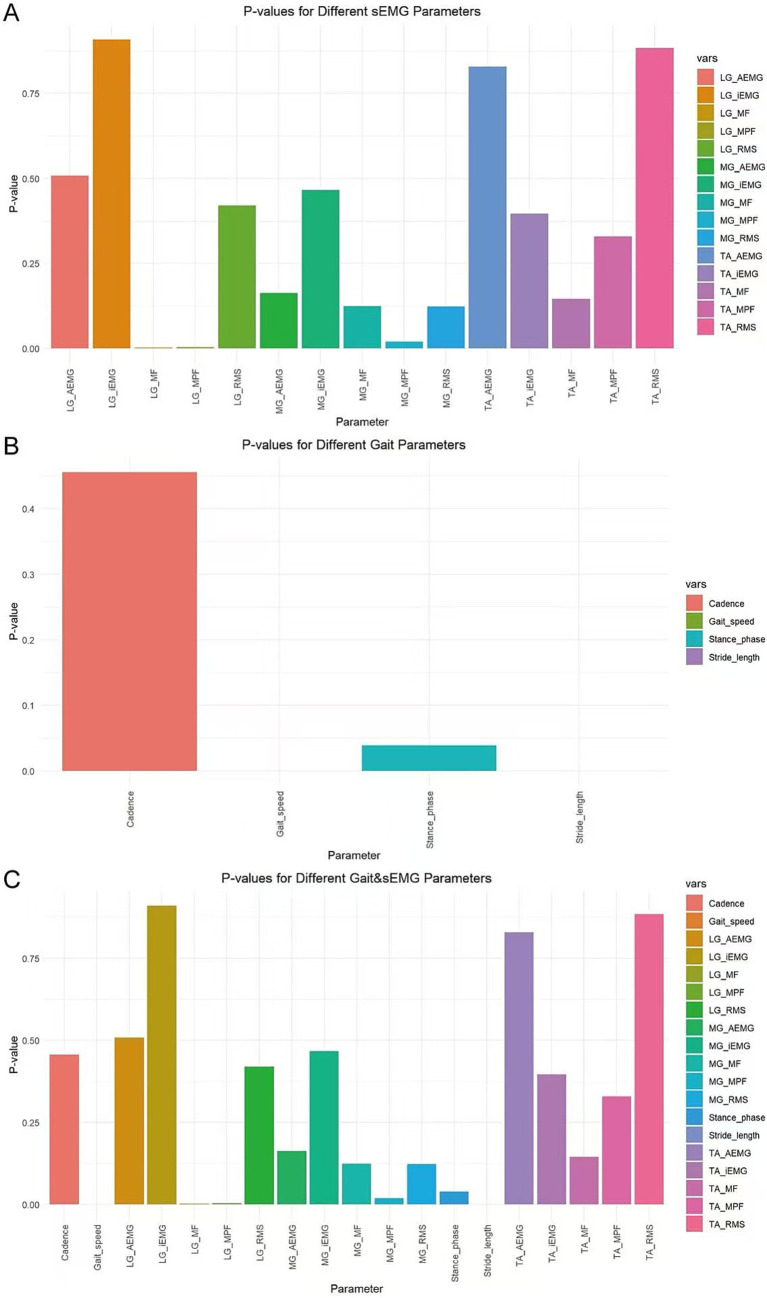
Variable selection for different datasets. The horizontal axis displays all variables of each dataset, while the vertical axis represents the *p*-value of each variable. **(A)** Variables and their associated *p*-values for the sEMG dataset **(B)** Variables and their associated p-values for the Gait dataset **(C)** Variables and their associated *p*-values for the Gait & sEMG dataset.

In this study, variables with p < 0.05 were screened using the ANOVA chi-square test method. For the sEMG dataset, the variables selected were mean power frequency of the medial gastrocnemius (MG_MPF), median frequency of the lateral gastrocnemius (LG_MF), and mean power frequency of the lateral gastrocnemius (LG_MPF) (Figure 5A). For the Gait dataset, the variables selected were Gait speed, Stride length, and Stance phase (Figure 5B). For the combined Gait & sEMG dataset, the variables selected were Gait speed, Stride length, Stance phase, MG_MPF, LG_MF, and LG_MPF (Figure 5C).

#### 3.2.2 Model performance evaluation

**Figure 6:**
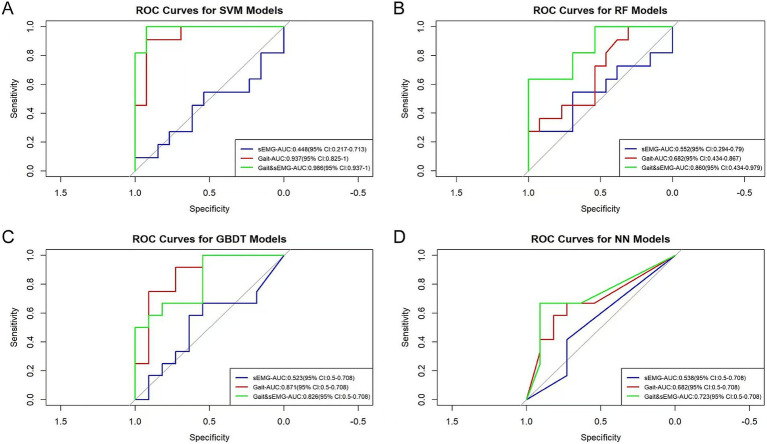
ROC curves of different models. The AUC values and 95% confidence intervals of different models are shown in the figure. **(A)** The ROC curves of the models built with SVM for three groups of datasets. The p-values for the sEMG, Gait, and Gait & sEMG datasets are 0.731, 0.074, and 0.001, respectively. **(B)** The ROC curves of the models built with RF for three groups of datasets. The p-values for the sEMG, Gait, and Gait & sEMG datasets are 0.271, 0.271, and 0.074, respectively. **(C)** The ROC curves of the models built with GBDT for three groups of datasets. The *p*-values for the sEMG, Gait, and Gait & sEMG datasets are 0.419, 0.026, and 0.071, respectively. **(D)** The ROC curves of the models built with NN for three groups of datasets. The *p*-values for the sEMG, Gait, and Gait & sEMG datasets are 0.998, 0.266, and 0.600, respectively.

Figure 6 shows the Receiver Operating Characteristic (ROC) curves for three datasets applying four algorithms. The AUC values of the sEMG dataset applying the SVM, RF, GBDT, and NN algorithms were 0.448, 0.552, 0.523, and 0.538, respectively, indicating poor model discrimination ability. The AUC values of the Gait dataset applying the SVM, RF, GBDT, and NN algorithms were 0.937, 0.682, 0.871, and 0.682, respectively, showing an improvement in model discrimination ability compared to the models constructed from the sEMG dataset. The combined Gait &sEMG dataset applying SVM, RF, GBDT, and NN algorithms had AUC values of 0.986, 0.860, 0.826, and 0.723, respectively, indicating a further enhancement in discrimination ability compared to the models constructed from the Gait dataset alone, except for the GBDT algorithm. Table 2 presents key model performance metrics. Of course, we also carried out the modeling process without variable selection (details are in the Supplementary material). Ultimately, we found that variable selection using the ANOVA chi-square test improved model performance. The model built with the SVM algorithm based on the Gait & sEMG dataset was optimal, with an AUC value as high as 0.986, indicating excellent discrimination ability.

**Table 2:** Comparison of key performance metrics of different models.

Algorithm	Dataset	Sensitivity	Specificity	Accuracy	Precision	Recall	F1-score
SVM	sEMG	0.727	0.308	0.500	0.471	0.727	0.571
	Gait	1.000	0.462	0.708	0.611	1.000	0.759
	Gait & sEMG	1.000	0.769	0.875	0.786	1.000	0.880
	sEMG	0.546	0.692	0.625	0.600	0.546	0.571
RF	Gait	0.909	0.385	0.625	0.556	0.909	0.690
	Gait & sEMG	0.727	0.692	0.708	0.667	0.727	0.696
	sEMG	0.546	0.583	0.565	0.545	0.545	0.545
GBDT	Gait	0.800	0.769	0.783	0.727	0.800	0.762
	Gait & sEMG	0.667	0.727	0.696	0.727	0.667	0.696
	sEMG	0.471	0.500	0.478	0.727	0.471	0.571
NN	Gait	0.615	0.700	0.652	0.727	0.615	0.667
	Gait & sEMG	0.625	0.857	0.696	0.909	0.625	0.741

### 3.3 Model optimization

On the basis of the sEMG, Gait, and Gait & sEMG datasets, we integrated baseline characteristics with *p* < 0.05 (age, height, and education duration) and imaging markers to construct the sEMG integration, Gait integration, and Gait & sEMG integration datasets, in order to optimize the models.

#### 3.3.1 Variable selection

**Figure 7:**
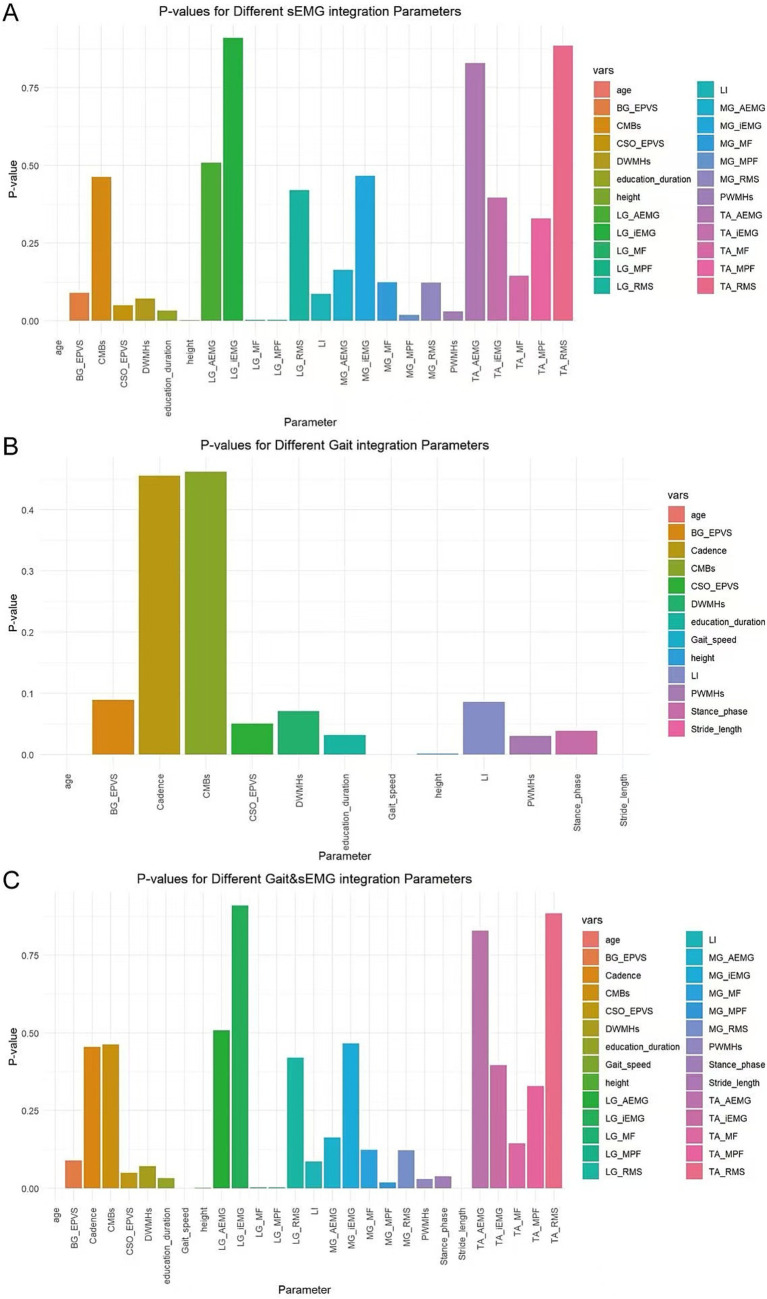
Variable selection for different integrated datasets. The horizontal axis displays all variables of each dataset, while the vertical axis represents the p-value of each variable. **(A)** Variables and their associated p-values for the sEMG integration dataset **(B)** Variables and their associated p-values for the Gait integration dataset **(C)** Variables and their associated p-values for the Gait & sEMG integration dataset.

In this study, variables with *p* < 0.05 were screened using the ANOVA chi-square test method. For the sEMG integration dataset, the variables selected were age, height, education duration, PWMHs, MG_MPF, LG_MF, and LG_MPF (Figure 7A). For the Gait integration dataset, the variables selected were age, height, education duration, PWMHs, Gait speed, Stride length, and Stance phase (Figure 7B). For the combined Gait & sEMG integration dataset, the variables selected were age, height, education duration, PWMHs, Gait speed, Stride length, Stance phase, MG_MPF, LG_MF, and LG_MPF (Figure 7C).

#### 3.3.2 Model performance evaluation

**Figure 8:**
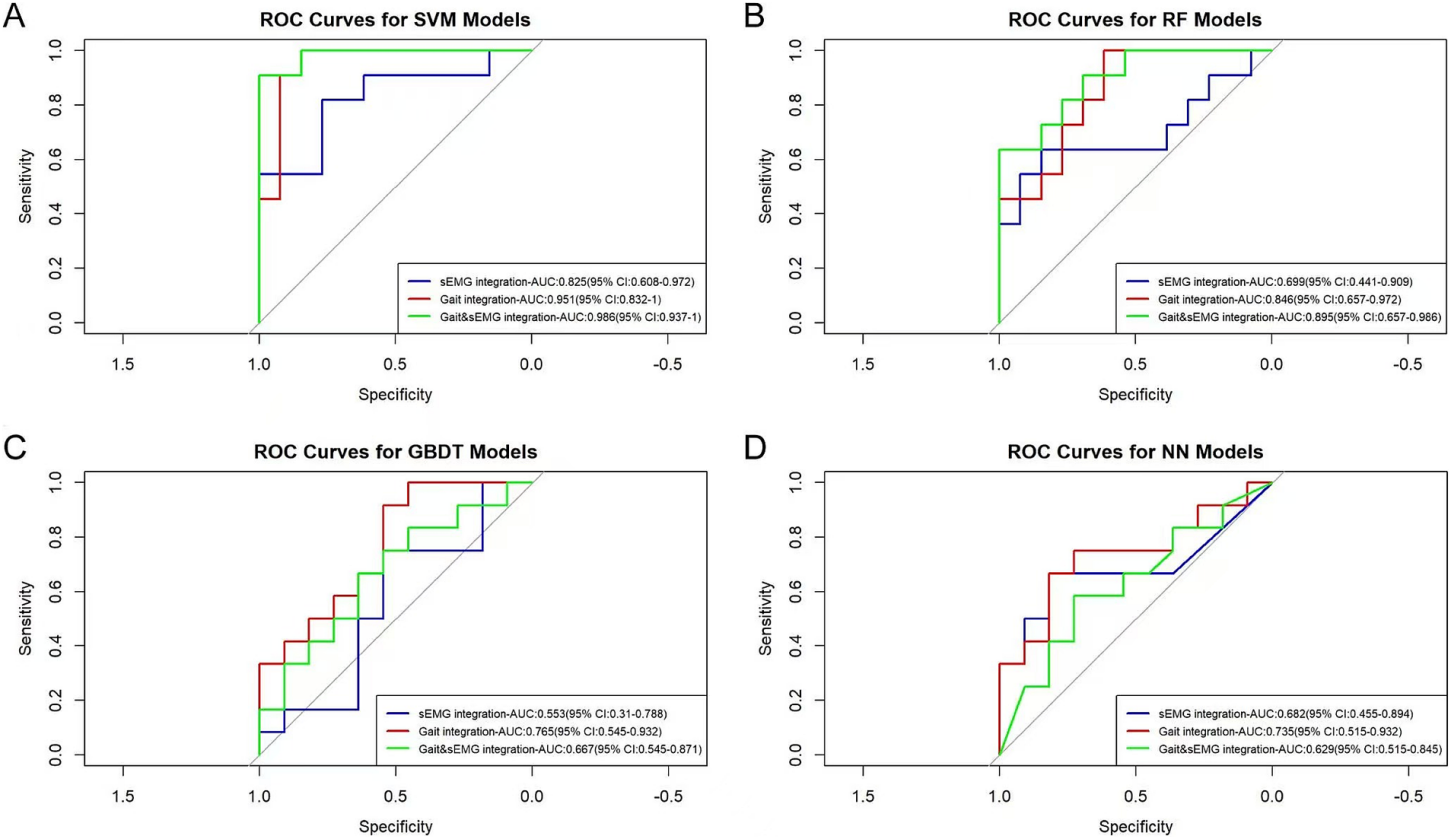
ROC curves of different optimized models. The AUC values and 95% confidence intervals of different optimized models are shown in the figure. **(A)** The ROC curves of the models built with SVM for three groups of integrated datasets. The p-values for the sEMG integration, Gait integration, and Gait & sEMG integration datasets are 0.074, 0.001, and 8.91E-05, respectively. **(B)** The ROC curves of the models built with RF for three groups of integrated datasets. The p-values for the sEMG integration, Gait integration, and Gait & sEMG integration datasets are 0.074, 0.010, and 0.030, respectively. **(C)** The ROC curves of the models built with GBDT for three groups of integrated datasets. The p-values for the sEMG integration, Gait integration, and Gait & sEMG integration datasets are 0.419, 0.590, and 0.267, respectively. **(D)** The ROC curves of the models built with NN for three groups of integrated datasets. The p-values for the sEMG integration, Gait integration, and Gait & sEMG integration datasets are 0.264, 0.422, and 0.267, respectively.

The AUC values for the sEMG integration dataset, when subjected to SVM, RF, GBDT, and NN algorithms, were 0.825, 0.699, 0.553, and 0.682, respectively. For the Gait integration dataset, the corresponding AUC values obtained from the application of these algorithms were 0.951, 0.846, 0.765, and 0.735, respectively. Furthermore, For the Gait & sEMG integration dataset, the corresponding AUC values obtained from the application of these algorithms were 0.986, 0.895, 0.667, and 0.629, respectively (Figure 8). These results indicate poor model discrimination ability with sEMG integration dataset, and the model built with the SVM algorithm based on the Gait & sEMG integration dataset was optimal, with an AUC value as high as 0.986. Table 3 presents key model performance metrics. For the integrated dataset, we also conducted the modeling process without variable selection (details are in the Supplementary material). Similarly, variable selection using ANOVA chi-square test can improve model performance, and the model built with the SVM algorithm based on the Gait & sEMG integration dataset was optimal. Although the AUC values of the models constructed using the SVM algorithm were the same for both the Gait & sEMG dataset and the Gait & sEMG integration dataset, a comparison of key performance metrics revealed that the model based on the Gait & sEMG integration dataset performed better.The model has high sensitivity (0.909) and specificity (0.923), indicating that it performs well in identifying both true positive and true negative cases of fall risk. These performance metrics, as assessed on the validation set, highlight the consistent and robust discriminatory power of the model across different datasets, implying that the model can effectively discriminate between covert CSVD patients with and without fall risk, suggesting that it may have important utility in clinical settings.

**Table 3:** Comparison of key performance metrics of different optimized models.

Algorithm	Integrated dataset	Sensitivity	Specificity	Accuracy	Precision	Recall	F1-score
SVM	sEMG	0.818	0.615	0.708	0.643	0.818	0.720
	Gait	1.000	0.769	0.875	0.786	1.000	0.880
	Gait & sEMG	0.909	0.923	0.917	0.909	0.909	0.909
	sEMG	0.546	0.846	0.708	0.750	0.546	0.632
RF	Gait	1.000	0.615	0.792	0.688	1.000	0.815
	Gait & sEMG	0.727	0.769	0.750	0.727	0.727	0.727
	sEMG	0.546	0.583	0.565	0.545	0.545	0.545
GBDT	Gait	0.571	0.667	0.609	0.727	0.571	0.640
	Gait & sEMG	0.583	0.636	0.609	0.636	0.583	0.609
	sEMG	0.643	0.778	0.696	0.818	0.643	0.720
NN	Gait	0.667	0.643	0.652	0.545	0.667	0.600
	Gait & sEMG	0.583	0.636	0.609	0.636	0.583	0.609

## 4 Discussion

In our research, a fall risk prediction model was successfully developed for patients with covert CSVD. The model utilizes wearable devices to acquire gait characteristics and sEMG features of CSVD patients, and combines selected baseline data with imaging markers, applying ML techniques for a comprehensive assessment of fall risk. This innovative methodology has enhanced the accuracy of fall risk prediction, offering significant insights into the evaluation of fall risk in CSVD, especially in the initial phases when clinical symptoms are subtle. Additionally, our results emphasize that the combination of gait characteristics, electromyographic features, baseline data, and imaging markers using the SVM algorithm is a reliable predictor of falls in CSVD, with an AUC of 0.986.

Walking is the result of coordination among multiple factors and systems, primarily due to an individual’s neuromuscular interaction abilities. Normal walking is maintained by integrating sensory signals from muscles, joints, and skin through the sensory system, which then inputs this information into the central nervous system (CNS). The CNS integrates this information and issues postural and balance control commands that are conducted via the vestibulospinal tract and corticospinal tracts, modulating muscle contractions and relaxation through nerve fibers to maintain body stability. In CSVD patients, the neural networks within the CNS responsible for movement and posture control are compromised, leading to impaired regulation of muscle contraction and relaxation, and a reduction in muscle strength. The TA and gastrocnemius muscles of the lower limbs play crucial roles in maintaining gait stability. The TA controls the speed of foot strike through eccentric contraction when the heel just touches the ground, ensuring the stability of foot landing. Additionally, during the pre-swing phase, the TA contracts, reducing ankle plantarflexion when the toes lift off the ground, ensuring the smooth completion of foot clearance. The gastrocnemius contracts during the terminal stance phase, propelling the body forward and upward, ensuring the transfer of body weight (Li et al., 2020). In CSVD patients, the decline of muscle strength in the lower limbs directly affects gait stability and increases the risk of falls. Spatiotemporal gait parameters such as stride length, gait speed, cadence, and double support phase are important indicators for assessing gait stability, and abnormalities in these parameters often indicate gait instability, thereby increasing the possibility of falls in patients (Li et al., 2020; Wang et al., 2023). Patients with CSVD typically exhibit gait disturbances characterized by shortened stride length, slowed gait speed, decreased cadence, and a prolonged double support phase (Kancheva et al., 2024; Mo et al., 2024; Mukli et al., 2022; Xu et al., 2024),all of which are clear signs of impaired gait stability and reduced balance ability (Mo et al., 2024; Mo et al., 2023; Shao et al., 2023; Sharma et al., 2023; Wang et al., 2023; Xu et al., 2024). The reduction in stride length as one of the early features of gait disturbances in CSVD patients, typically associated with diminished propulsive force due to decreased muscle strength, which can subsequently lead to a decline in gait speed. Reduced gait speed not only reflects the decline of individual functional status but also serves as an early signal of gait impairment, suggesting an underlying system compromise (Studenski et al., 2011; Wang et al., 2023).In addition, patients with CSVD may also exhibit a reduced cadence and an increased percentage of the double support phase, which are considered compensatory mechanisms for gait instability, aiming to improve stability, prevent falls, or reduce the energy cost of activity. However, when a patient progresses to the decompensated phase of the disease, the risk of falls escalates significantly. Therefore, spatiotemporal parameters associated with fall risk were included in this research to improve the prediction of fall risk.

Falls can lead to a cascade of adverse outcomes, particularly for the elderly demographic, who are susceptible to serious consequences such as increased mortality risk, reduced quality of life, pain, increased hospitalization, and long-term lifestyle alterations (Del Din et al., 2019; Subramaniam et al., 2022). Beyond the immediate physical repercussions, the fear of falling can significantly disrupt a patient’s routine activities and impede the pursuit of a healthy lifestyle (Subramaniam et al., 2022). Predicting, screening and preventing falls in patients with CSVD who may face a significant risk for future gait disorders and fall events is a priority, but it remains a formidable challenge for healthcare providers (Chen et al., 2022). Although a history of falls is the most reliable predictor of future falls (van Schooten et al., 2015), it has limited effectiveness for the early identification and intervention of initial fall events (Bargiotas et al., 2023). Currently, there is still a lack of predictive models for fall risk based on gait monitoring systems in CSVD patients, which restricts our capacity for early detection and timely intervention.

As wearable technology continues to evolve, real-time gait monitoring has become possible. These technologies enable data collection in natural and daily activity settings and have been utilized for fall detection and fall risk assessment (Subramaniam et al., 2022; Wuehr et al., 2021). Gait analysis, an alternative approach to identify fall risk in the elderly (Chen et al., 2022; García-de-Villa et al., 2023; Lonini et al., 2022), has seen increased usage over the past few decades. Inertial Measurement Units (IMUs) have shown good consistency with traditional gait analysis in certain spatiotemporal parameters, enabling the monitoring and analysis of gait characteristics, and several IMU-based fall prediction factors have been introduced (Berner et al., 2020; Nishiyama et al., 2024; Ruiz-Ruiz et al., 2021; Saadeh et al., 2019; Yıldız, 2023).The rapid advancement of ML has significantly improved fall risk prediction and classification through the integration of ML with gait characteristics captured by inertial measurement units (IMUs) (Subramaniam et al., 2022). Additionally, sEMG, known for its ability to reflect the subtle manifestations of abnormal gait, has been widely used in identifying and forecasting FOG in Parkinson’s disease. However, its application has been limited in studies of gait disturbances and fall risk associated with CSVD. Further investigation in this field is needed to advance knowledge of the underlying mechanisms and their clinical implications. To confront this challenge, our study employed portable sEMG for data collection during the natural ambulation of subjects and applied it to predict fall risk, significantly expanding and enhancing the predictive model from a microstructural and deeper mechanism perspective. Moreover, Feature capture under dual-task conditions more closely resembles daily activities, laying the foundation for assessing fall risk in natural environments.

Our study presents several limitations. Firstly, while cognitive impairment is known for its complex relationship with gait disturbances, potentially increasing the risk of falls (Dubbioso et al., 2023; Pieruccini-Faria et al., 2020), our research did not incorporate established measures of cognitive function such as the Mini-Mental State Examination (MMSE) or Montreal Cognitive Assessment (MoCA), despite examining gait and electromyographic data during tasks that required cognitive engagement. Although cognition was not the primary focus, its influence on gait dynamics and fall incidents underscores the complexity of mechanisms underlying gait disorders and falls, highlighting the need for a broader consideration of contributing factors. Furthermore, a wide range of factors, such as physiological, psychological, and environmental ones, can affect falls and either work alone or in concert to increase an individual’s vulnerability to falls (Subramaniam et al., 2022). The psychological and environmental factors not considered in this study will be the direction of our subsequent research. In terms of gait characteristics, our research selected a range of commonly used spatiotemporal gait parameters, which are crucial for providing insights into critical gait attributes such as stride length and gait speed. Additionally, we included time-domain and frequency-domain indicators closely related to muscle activity and fatigue. These indicators provide valuable insights into muscle function and its effect on gait stability. However, we did not explore kinematic parameters or nonlinear sEMG metrics, which could offer further insights into joint dynamics and muscle activity. Unfortunately, no sensors were worn during the TUG examination in this study because the wearable SRE, equipped with shank and foot brackets, is prone to collisions. These collisions, particularly during the turning phase of the TUG test, may lead to falls and affect the results. The development of brackets that more closely conform to the shank and foot is key to resolving this issue. Although the SRE can monitor gait information in real-time, patients and untrained physicians still face some difficulty in wearing it correctly. Further development of a smaller, easier-to-wear integrated power supply and brackets may enhance patient compliance.

Despite these limitations, our study still holds significant importance, offering clinicians a simple and effective tool to identify fall risk in covert CSVD. These limitations do not diminish its contribution to clinical practice and future research. In fact, they provide direction for future work, indicating that our future research should consider employing more convenient devices to capture gait information and should account for more factors affecting falls, such as cognition and psychological factors, and should incorporate more gait assessment indicators. This will contribute to developing a more comprehensive and precise tool for assessing fall risk in patients with covert CSVD and provide a stronger scientific basis for formulating targeted intervention measures.

## 5 Conclusion

In conclusion, our research offers a useful method for identifying fall risk early in those with covert CSVD. Although the study has certain limitations, such as the non-consideration of cognitive scales, psychological factors and environmental elements, which may exert some influence on the results, these limitations do not detract from its significant contribution to clinical practice. This tool has the potential to significantly enhance clinical decision-making and prognostication, thereby reinforcing the efficacy of healthcare practices.

## Data Availability

The raw data supporting the conclusions of this article will be made available by the authors, without undue reservation.
