# Supplementary material for: Wearable sensors and machine learning fusion-based fall risk prediction in covert cerebral small vessel disease
Source: Front Neurosci. 2025 Feb 19;19:1493988. doi: 10.3389/fnins.2025.1493988 (PMC11879974; doi:10.3389/fnins.2025.1493988)
Supplement: Supplementary file 1 [file Data_Sheet_1.DOCX]

The AUC values for the sEMG dataset, when subjected to SVM, RF, GBDT, and NN algorithms, were 0.531, 0.580, 0.659, and 0.545, respectively. For the Gait dataset, the corresponding AUC values obtained from the application of these algorithms were 0.832, 0.741, 0.864, and 0.439, respectively. Furthermore, For the Gait & sEMG dataset, the corresponding AUC values obtained from the application of these algorithms were 0.853, 0.902, 0.811, and 0.451, respectively (Figure S1). Table S1 presents key model performance metrics. Among the twelve models constructed without screening variables, the model constructed by applying the RF algorithm based on the Gait & sEMG dataset was relatively better, with an AUC value of 0.902, a sensitivity of 0.818, and a specificity of 0.692. It was inferior to the models constructed after variable selection using the chi-square test method, both in terms of model discrimination ability and in identifying true positive and true negative cases of fall risk.


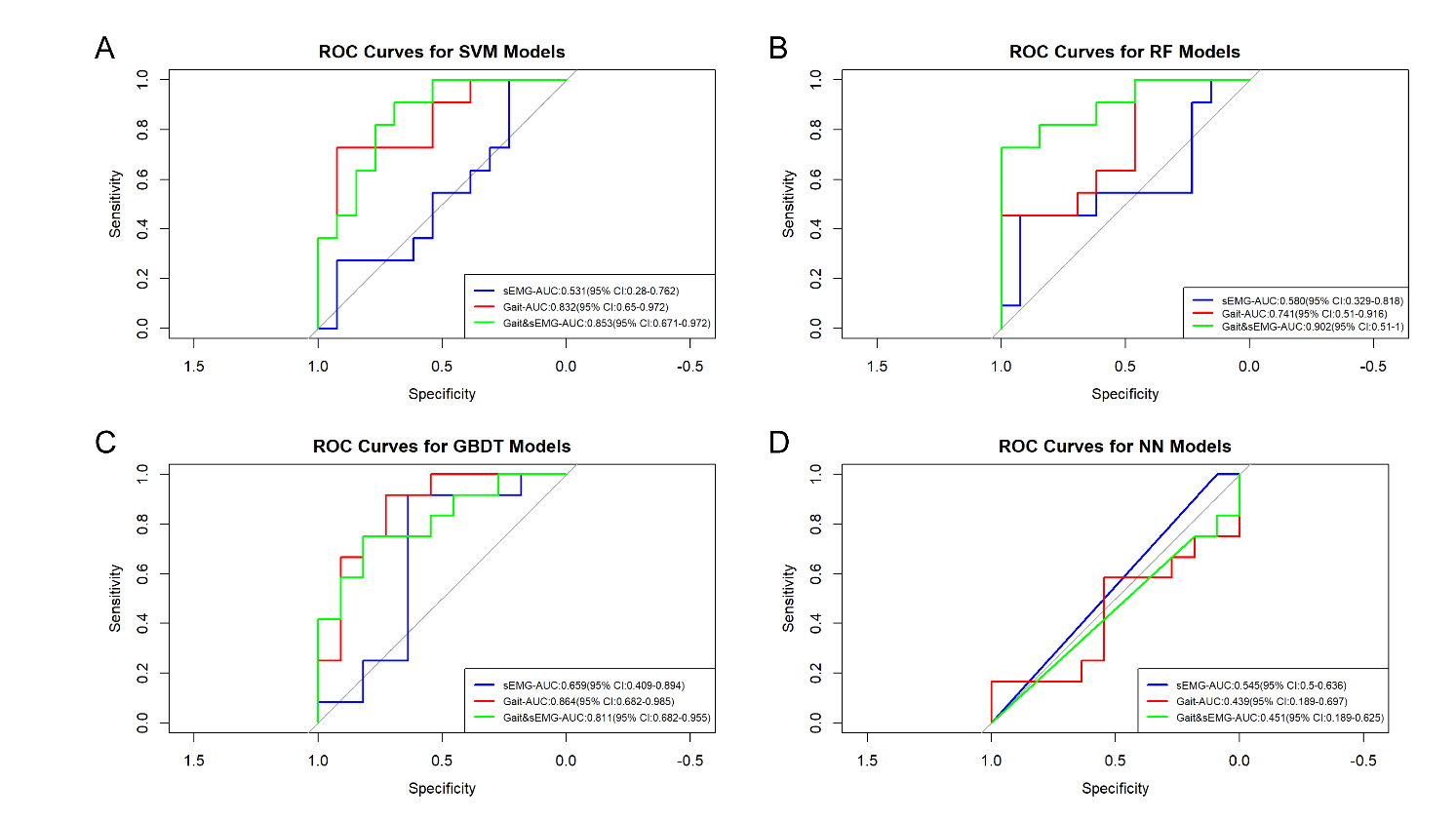


**Supplementary Figure 1.** ROC curves of different models. The AUC values and 95% confidence intervals of different models are shown in the figure. (A) The ROC curves of the models built with SVM for three groups of datasets. The P-values for the sEMG, Gait, and Gait & sEMG datasets are 0.847, 0.153, and 0.074, respectively. (B) The ROC curves of the models built with RF for three groups of datasets. The P-values for the sEMG, Gait, and Gait & sEMG datasets are 0.847, 0.153, and 0.030, respectively. (C) The ROC curves of the models built with GBDT for three groups of datasets. The P-values for the sEMG, Gait, and Gait & sEMG datasets are 0.973, 0.026, and 0.260, respectively. (D) The ROC curves of the models built with NN for three groups of datasets. The P-values for the sEMG, Gait, and Gait & sEMG datasets are 0.584, 0.267, and 0.852, respectively.

**Supplementary Table1.** Comparison of key performance metrics of different models

| Algorithm | Dataset | sensitivity | specificity | accuracy | precision | recall | F1-score |
| --- | --- | --- | --- | --- | --- | --- | --- |
|  | sEMG | 1.000 | 0.000 | 0.458 | 0.458 | 1.000 | 0.629 |
| SVM | Gait | 0.909 | 0.462 | 0.667 | 0.588 | 0.909 | 0.714 |
|  | Gait & sEMG | 0.909 | 0.539 | 0.708 | 0.625 | 0.909 | 0.741 |
|  | sEMG | 0.546 | 0.385 | 0.458 | 0.429 | 0.546 | 0.480 |
| RF | Gait | 1.000 | 0.385 | 0.667 | 0.579 | 1.000 | 0.733 |
|  | Gait & sEMG | 0.818 | 0.692 | 0.750 | 0.692 | 0.818 | 0.750 |
|  | sEMG | 0.467 | 0.500 | 0.478 | 0.636 | 0.467 | 0.538 |
| GBDT | Gait | 0.800 | 0.769 | 0.783 | 0.727 | 0.800 | 0.762 |
|  | Gait & sEMG | 0.667 | 0.875 | 0.739 | 0.909 | 0.667 | 0.769 |
|  | sEMG | 0.000 | 1.000 | 0.522 | 0.522 | 1.000 | 0.686 |
| NN | Gait | 0.364 | 0.833 | 0.609 | 0.588 | 0.833 | 0.690 |
|  | Gait & sEMG | 0.091 | 0.750 | 0.435 | 0.474 | 0.750 | 0.581 |

The AUC values for the sEMG integration dataset, when subjected to SVM, RF, GBDT, and NN algorithms, were 0.804, 0.678, 0.583, and 0.545, respectively. For the Gait integration dataset, the corresponding AUC values obtained from the application of these algorithms were 0.944, 0.867, 0.727, and 0.625, respectively. Furthermore, For the Gait & sEMG integration dataset, the corresponding AUC values obtained from the application of these algorithms were 0.923, 0.937, 0.636, and 0.500, respectively (Figure S2). Table S2 presents key model performance metrics. Among the twelve models constructed without screening variables, the model constructed by applying the SVM algorithm based on the Gait integration dataset was relatively better, with an AUC value of 0.944, a sensitivity of 1.000, and a specificity of 0.462. It was inferior to the models constructed after variable selection using the chi-square test method, both in terms of model discrimination ability and in identifying true positive and true negative cases of fall risk.


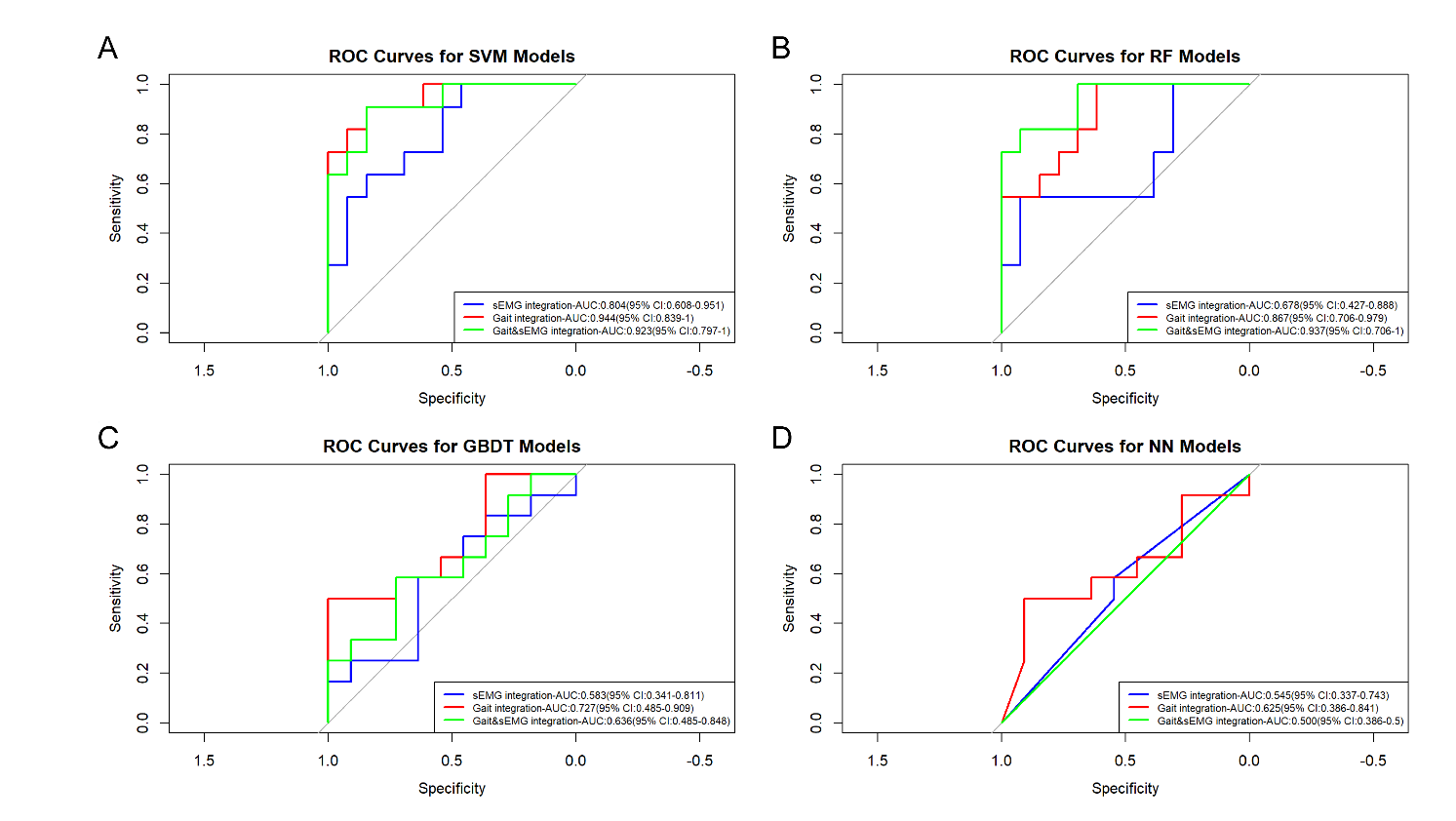


**Supplementary Figure 2.** ROC curves of different optimized models. The AUC values and 95% confidence intervals of different optimized models are shown in the figure. (A) The ROC curves of the models built with SVM for three groups of integrated datasets. The P-values for the sEMG integration, Gait integration, and Gait & sEMG integration datasets are 0.583, 0.074, and 0.153, respectively. (B) The ROC curves of the models built with RF for three groups of integrated datasets. The P-values for the sEMG integration, Gait integration, and Gait & sEMG integration datasets are 0.421, 0.030, and 0.010, respectively. (C) The ROC curves of the models built with GBDT for three groups of integrated datasets. The P-values for the sEMG integration, Gait integration, and Gait & sEMG integration datasets are 0.587, 0.590, and 0.863, respectively. (D) The ROC curves of the models built with NN for three groups of integrated datasets. The P-values for the sEMG integration, Gait integration, and Gait & sEMG integration datasets are 0.584, 0.071, and 0.584, respectively.

**Supplementary Table2.** Comparison of key performance metrics of different optimized models

| Algorithm | Integrated dataset | sensitivity | specificity | accuracy | precision | recall | F1-score |
| --- | --- | --- | --- | --- | --- | --- | --- |
|  | sEMG | 1.000 | 0.154 | 0.542 | 0.500 | 1.000 | 0.667 |
| SVM | Gait | 1.000 | 0.462 | 0.708 | 0.611 | 1.000 | 0.759 |
|  | Gait & sEMG | 1.000 | 0.385 | 0.667 | 0.579 | 1.000 | 0.733 |
|  | sEMG | 0.546 | 0.615 | 0.583 | 0.546 | 0.546 | 0.546 |
| RF | Gait | 0.909 | 0.615 | 0.750 | 0.667 | 0.909 | 0.769 |
|  | Gait & sEMG | 0.818 | 0.769 | 0.792 | 0.750 | 0.818 | 0.783 |
|  | sEMG | 0.539 | 0.600 | 0.565 | 0.636 | 0.538 | 0.583 |
| GBDT | Gait | 0.571 | 0.667 | 0.609 | 0.727 | 0.571 | 0.640 |
|  | Gait & sEMG | 0.533 | 0.625 | 0.565 | 0.727 | 0.533 | 0.615 |
|  | sEMG | 0.546 | 0.500 | 0.522 | 0.545 | 0.500 | 0.522 |
| NN | Gait | 0.909 | 0.500 | 0.696 | 0.857 | 0.500 | 0.632 |
|  | Gait & sEMG | 0.000 | 1.000 | 0.522 | 0.522 | 1.000 | 0.686 |
